# Extended Bacteria Culture-Based Clustering Identifies a Phenotype Associating Increased Cough and *Enterobacterales* in Stable Chronic Obstructive Pulmonary Disease

**DOI:** 10.3389/fmicb.2021.781797

**Published:** 2021-12-14

**Authors:** Anaëlle Muggeo, Jeanne-Marie Perotin, Audrey Brisebarre, Sandra Dury, Valérian Dormoy, Claire Launois, Julien Ancel, Pauline Mulette, Christophe de Champs, Gaëtan Deslée, Thomas Guillard

**Affiliations:** ^1^Inserm UMR-S 1250 Pulmonary pathologies and cellular plasticity (P3Cell), Reims-Champagne-Ardenne University, SFR CAP Santé, Reims, France; ^2^Laboratory of Bacteriology-Virology-Hospital Hygiene-Parasitology-Mycology, Reims University Hospital, Reims, France; ^3^Department of Respiratory Diseases, Reims University Hospital, Reims, France

**Keywords:** COPD—chronic obstructive pulmonary disease, *Enterobacterales*, microbiota, extended culture, stable state

## Abstract

Chronic obstructive pulmonary disease (COPD) is a chronic inflammatory lung disease characterized by airflow limitation. This chronic respiratory disease represents the third leading cause of death worldwide. Alteration of the airway microbiota has been reported to be associated with exacerbation frequency in COPD, but its role on the symptoms in patients at stable state is still incompletely described. This study aimed to determine whether bacteria isolated in sputum can be associated with the clinical features of COPD patients within stable state. Our study highlights, for the first time, that altered microbiota with *Enterobacterales* is associated with pejorative clinical symptoms in stable COPD patients. The airway microbiota of 38 patients was analyzed using an extended culture approach and mass spectrometry identification. Cluster analysis by principal coordinate analysis of the bacterial communities showed that the patients could be classified into three distinct clusters in our cohort. The clusters showed no differences in proportions of the phylum, but one of them was associated with a high prevalence of *Enterobacterales* (71.4% in cluster 1 vs. 0% in cluster 3), loss of microbiota diversity, and higher bacterial load (10^7^ vs. 10^5^ CFU/ml, respectively) and characterized by predominant cough and impact on mental health. These novel findings, supported by further studies, could lead to modifying the processing of COPD sputum in the everyday practice of clinical microbiology laboratories.

## Introduction

Chronic obstructive pulmonary disease (COPD) is a heterogeneous disease characterized by progressive airflow limitation, associating airway inflammation and remodeling, and lung parenchymal destruction. The pathophysiological mechanisms involved in COPD development are still not completely elucidated. COPD represents a major worldwide health challenge regarding morbidity and mortality, facing substantial and increasing economic and social burden, with a limited effect of current treatments (Global Initiative for Chronic Obstructive Lung Disease, 2021). Although highly variable, COPD-related symptoms usually associate chronic cough, sputum, and dyspnea on exercise. Acute exacerbations (AE-COPD), which are defined as acute worsening of respiratory symptoms requiring treatment modifications, are of high impact in terms of immediate morbidity and mortality and long-term COPD worsening (Global Initiative for Chronic Obstructive Lung Disease, 2021). AE-COPD are mainly due to bacterial airway proliferation, such as *Haemophilus influenzae*, *Streptococcus pneumoniae*, *Moraxella catarrhalis*, *Pseudomonas aeruginosa*, and *Staphylococcus aureus* (Sethi and Murphy, 2008; Matkovic and Miravitlles, 2013; Leung et al., 2017). In addition to their role in AE-COPD, these potential pathogenic microorganisms (PPM), which have not been described in healthy lungs (Sethi and Murphy, 2008), have been detected in the airways of stable COPD patients with no sign of AE-COPD (Leung et al., 2017). In the light of the description of the lung as a non-sterile organ (Dickson et al., 2014; Moffatt and Cookson, 2017; Martin-Loeches et al., 2020), a demonstration of complex polymicrobial communities in the lower respiratory tract (Hilty et al., 2010; Huang et al., 2010; Erb-Downward et al., 2011; Cabrera-Rubio et al., 2012) has questioned whether alteration of the endogenous microbiota may impact COPD pathogenesis. Interestingly, some studies showed that the increasing load of bacteria in the airways of stable COPD patients was associated with exacerbation frequency and airflow limitation (Patel et al., 2002; Wilkinson et al., 2003; Garcha et al., 2012; Galiana et al., 2013).

Advances in DNA sequencing allowed an extensive description of the lung microbiota in COPD patients. Studies providing exhaustive descriptions of lung bacterial communities depending on COPD features mainly focused on dysbiosis in AE-COPD (Huang et al., 2010, 2014; Su et al., 2015; Filho et al., 2018; Ghebre et al., 2018). Moreover, contradictory studies that are using such molecular approaches reported either a decrease or an increase of the microbiota diversity (Pragman et al., 2012; Sze et al., 2015). Whether the alterations of the microbiota in stable COPD patients are associated with AE-COPD onset is currently not known.

Airway microbiota analyses in stable COPD also showed inconsistent results (Garcia-Nuñez et al., 2014; Diao et al., 2018; Wang et al., 2018; Tangedal et al., 2019; Dicker et al., 2021; Yang et al., 2021), highlighting a predominance of *Streptococcus* spp. (Tangedal et al., 2019; Yang et al., 2021) or *Neisseria* spp. (Diao et al., 2018). Some studies suggested a link between the community composition or the microbiota diversity and the severity of the disease. However, the relationships between symptoms and airway dysbiosis at a stable state are still incompletely described (Galiana et al., 2013; Garcia-Nuñez et al., 2014; Diao et al., 2018). Notably, a decrease in diversity, with a loss of the resident flora which is replaced by a more restricted microbiota including PPM, has been reported in the most severe stable COPD patients (Galiana et al., 2013; Garcia-Nuñez et al., 2014). The heterogeneous clinical presentations observed in COPD could be explained, in part, by the type of airway microbiota at a stable state.

Most of the studies aiming at describing the airway microbiota used culture-independent techniques, such as PCR amplification and sequencing of the bacterial 16S ribosomal RNA gene (Pragman et al., 2012; Garcia-Nuñez et al., 2014; Wang et al., 2016; Diao et al., 2018; Jubinville et al., 2018; Mayhew et al., 2018; Tangedal et al., 2019; Dicker et al., 2021; Yang et al., 2021). Conversely to the 16S rRNA sequencing, conventional culture-based approaches have the great advantage to be applicable in any clinical microbiology laboratory using MALDI-TOF technologies, which are now very commonly used for identification (Dingle and Butler-Wu, 2013).

In this study, we decided to use such a strategy to identify viable airway microbiota and determine whether bacteria isolated in the sputum of COPD patients at a stable state is associated with relevant clinical features.

## Materials and Methods

### Study Population

COPD patients were prospectively included in the Recherche et INNOvation en PAthologie Respiratoire Inflammatoire (RINNOPARI) cohort (University Hospital of Reims, France; NCT02924818). The study was approved by the regional ethics committee (Comité de Protection des Personnes—Dijon EST I, no. 2016-A00242-49). Informed consent was obtained from all the patients.

Patients with asthma, cystic fibrosis (CF), bronchiectasis, or pulmonary fibrosis were excluded. Patients were included at a stable state (at least 4 weeks from the last exacerbation). At inclusion, the following characteristics were recorded: demographic data, smoking history, inhaled treatment, respiratory symptoms [modified Medical Research Council dyspnea scale, cough and sputum assessment questionnaire (CASA-Q) containing four domains (cough symptoms, cough impact, sputum symptoms, and sputum impact) scored from 0 to 100 with higher scores associated with fewer symptoms or less impact, COPD assessment test (CAT score) assessing the global impact of COPD on health status, exacerbation history, hospital anxiety and depression score, quality of life (St. George’s Respiratory Questionnaire, and 36-item short-form health survey (SF-36)], arterial blood gas, 6-min walking distance, and pulmonary function test results. COPD was defined by postbronchodilator FEV_1_/FVC < 70%. The severity of COPD was determined by spirometric classification (GOLD 1: FEV_1_ ≥ 80%; GOLD 2: 50% ≤ FEV_1_ < 80%; GOLD 3: 30% ≤ FEV_1_ < 50%; GOLD 4: FEV_1_ < 30%). CT scan images were analyzed by two independent investigators (SD and GD) using visual emphysema quantification (Washko et al., 2009; Perotin et al., 2014).

### Microbiological Analysis

Non-induced sputum was collected at inclusion, and an extended microbiological culture was performed, as previously described (Sherrard et al., 2019; Einarsson et al., 2021). Sputum quality assessment was performed for each sputum according to the Bartlett score (Bartlett et al., 1998). Sputa contaminated with saliva (squamous epithelial cells >25 per field) were discarded. Since stable COPD patients are likely to present little inflammation or active infection, cultures were performed for sputum with 10–25 or >25 leukocytes per field (inflammation) and also with less than 10 leukocytes per field (no inflammation). The extended culture consisted of a more complex culture than the conventional sputum culture: we added more media (including selective media), more atmospheres (including anaerobic culture), and more dilutions to detect bacteria with low load. Serial dilutions (1/1,000, 1/10,000, and 1/100,000) of the sputum were made after liquefaction by N-acetylcysteine and were cultured in Columbia blood agar, chocolate agar, Schaedler agar, and *Pseudomonas*-selective cetrimide agar (Thermo Fisher Scientific, United States) at 37°C for 48 h for aerobic cultures and 5% CO_2_ and 5 days for anaerobic cultures (Supplementary Table 1). All colonies that appeared to be morphologically distinct were quantified as colony-forming unit (CFU) per milliliter and identified by MALDI-TOF mass spectrometry (MALDI Biotyper^®^, Bruker Daltonics, Bremen, Germany). The α-diversity of the airway microbiota was evaluated with the Shannon index (a marker of intra-individual diversity).

### Statistical Analysis

The data are expressed as mean values ± standard deviation, median (interquartile range), or numbers and percentages when appropriate. Comparisons were performed using chi-square test or Fisher exact test for qualitative variables and *t*-test or Mann–Whitney test for quantitative variables when appropriate. Kruskal–Wallis test with Dunn’s multiple-comparison test was used to compare the three clusters. A *p*-value < 0.05 was considered significant.

To explore and visualize, in a low-dimensional Euclidean space, dissimilarities in bacterial communities between groups, unsupervised principal coordinate analysis (PCoA) was plotted using the Bray–Curtis dissimilarity matrix. We applied unsupervised clustering with a k-means algorithm on the distance data to distinguish three clusters of patients. Spanning ellipsoid was calculated for each cluster of points with the ellipsoidhull function in the R cluster package.

## Results

### Airway Microbiota Cluster Analysis

**FIGURE 1:**
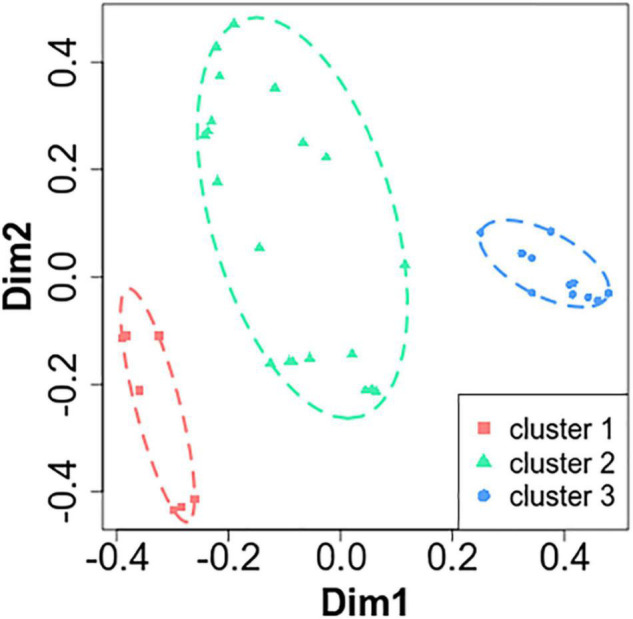
Principal coordinate analysis (PCoA) on microbiota revealed three clusters in stable chronic obstructive pulmonary disease patients. Unsupervised PcoA was plotted based on the Bray–Curtis dissimilarity matrix. The two most important eigenvectors were used for visualization (19 and 13% of variance explained, respectively). Clusters of patients were identified based on the k-means algorithm, and a spanning ellipsoid was added.

We prospectively included 38 COPD patients at a stable state (Table 1). The viable airway microbiota of the 38 sputa (one for each patient) was determined by extended culture. Globally, we identified 261 bacteria from 60 different species, representing a mean of 6.9 bacteria per sample. We then performed unbiased clustering using PCoA, depending on airway microbiota similarities between patients, which identified three clusters of patients (Figure 1): cluster 1 (*n* = 7, 18.4%), cluster 2 (*n* = 20, 52.6%), and cluster 3 (*n* = 11, 28.9%).

**TABLE 1:** Characteristics of the patients in the total group and the clusters based on airway microbiota.

	Total	Cluster 1	Cluster 2	Cluster 3	*p*-value		
					Cluster 1 *vs*. 3	Cluster 1 *vs*. 2	Cluster 2 *vs*. 3
Number	38	7	20	11			
Male	24 (63.2%)	2 (28.6%)	16 (80.0%)	6 (54.5%)	0.280	0.013	0.135
Age, years	60.9 ± 9.4	61.4 ± 11.5	59.2 ± 9.2	63.5 ± 8.5	0.659	0.609	0.205
BMI, kg/m^2^	26.0 ± 6.2	22.2 ± 4.1	27.4 ± 6.5	25.9 ± 6.1	0.182	0.063	0.544
**Smoking history**							
Former	25 (65.8%)	5 (71.4%)	12 (60.0%)	8 (72.7%)	0.952	0.590	0.479
Pack-years	47.3 ± 18.4	54.9 ± 31.5	45.1 ± 10.8	46.5 ± 19.5	0.492	0.2287	0.797
**Exacerbations in the last year**							
Number of patients	26 (68.4%)	5 (71.4%)	14 (70.0%)	7 (63.6%)	0.724	0.943	0.717
Number of episodes per patient	2.4 ± 1.4	2.8 ± 1.6	2.3 ± 1.1	2.4 ± 1.8	0.732	0.450	0.826
**Symptoms**							
Cough	29 (76.3%)	7 (100%)	16 (80.0%)	6 (54.5%)	**0.036**	0.264	0.077
Dyspnea ≥2 mMRC	31 (81.5%)	7 (100%)	15 (75.0%)	9 (81.8%)	0.497	0.283	1
Chronic bronchitis	18 (47.4%)	5 (71.4%)	8 (40.0%)	5 (45.5%)	0.280	0.152	0.768
CAT total score	19.0 ± 7.8	23.8 ± 4.6	18.2 ± 7.6	17.8 ± 9.3	0.204	0.134	0.895
**Lung function**							
FEV_1_, % pred	45.5 ± 19.5	48.2 ± 8.1	51.8 ± 21.9	46.7 ± 19.0	0.797	0.726	0.549
FEV_1_/FVC	47.6 ± 12.0	46.0 ± 5.7	49.8 ± 11.3	47.8 ± 13.5	0.621	0.490	0.695
RV, % pred	215.7 ± 90.5	243.3 ± 60.1	213.9 ± 104.8	201.0 ± 85.5	0.273	0.497	0.736
GOLD 3-4	22 (57.9%)	6 (85.7%)	10 (50.0%)	7 (63.6%)	0.596	0.183	0.707
6-min walking distance, n	32	7	16	9			
Distance, % of predicted value	66.4 ± 22.6	69.3 ± 16.6	68.8 ± 26.6	60.1 ± 19.8	0.341	0.945	0.466
CT scan, *n*	35	7	19	9			
Emphysema, *n*	29 (82.9%)	7 (100%)	14 (73.7%)	8 (88.9%)	0.362	0.131	0.36
Emphysema visual score	9.2 ± 4.7	7.0 ± 2.4	10.4 ± 5.3	9.1 ± 4.6	0.299	0.124	0.570
Questionnaires, *n*	37	7	20	10			
**CASA-Q**							
Cough symptoms	64.2 ± 21.7	51.2 ± 32.8	63.7 ± 16.3	74.2 ± 19.0	0.087	0.194	0.129
Sputum symptoms	58.8 ± 24.1	53.6 ± 32.2	52.9 ± 21.0	74.2 ± 18.6	0.115	0.951	0.011
Cough impact	70.1 ± 24.1	50.0 ± 18.0	70.5 ± 22.2	83.4 ± 23.6	**0.007**	0.038	0.151
Sputum impact	74.2 ± 29.9	58.3 ± 22.7	73.5 ± 19.01	86.7 ± 16.5	**0.009**	0.095	0.074
**HAD**							
Anxiety score	8.4 ± 4.5	12.2 ± 4.4	8.2 ± 4.0	6.6 ± 4.7	**0.034**	0.044	0.348
Depression score	6.6 ± 3.9	6.5 ± 3.3	6.7 ± 4.6	6.5 ± 2.8	1	0.941	0.925
**SGRQ**							
Total	52.9 ± 19.0	57.6 ± 14.9	52.4 ± 20.6	50.7 ± 19.3	0.440	0.548	0.826
**SF-36**							
Global physical health	33.5 ± 10.4	35.2 ± 12.4	33.1 ± 9.0	33.1 ± 12.6	0.742	0.633	0.992
Global mental health	31.3 ± 18.2	15.6 ± 19.2	33.4 ± 17.7	37.9 ± 13.0	**0.012**	0.034	0.485

*Unless otherwise stated, data are available for all patients. Indicated in bold are the characteristics that are statistically significant between clusters 1 and 3 (chi-square test, Fisher exact test, t-test, and Mann–Whitney test).*

*BMI, body mass index; mMRC, modified Medical Research Council dyspnea scale; FEV, forced expiratory volume in 1 s; FVC, forced vital capacity; RV, residual volume; CASA-Q, Cough and Sputum Assessment Questionnaire; HAD, Hospital Anxiety and Depression Scale; SGRQ, St. George’s Respiratory Questionnaire; SF-36, 36-item short-form health survey.*

### Airway Microbiota Comparison in the Three Clusters

To identify the key microbial drivers of the clustering, we first compared the microbiota of the three clusters of COPD patients.

**FIGURE 2:**
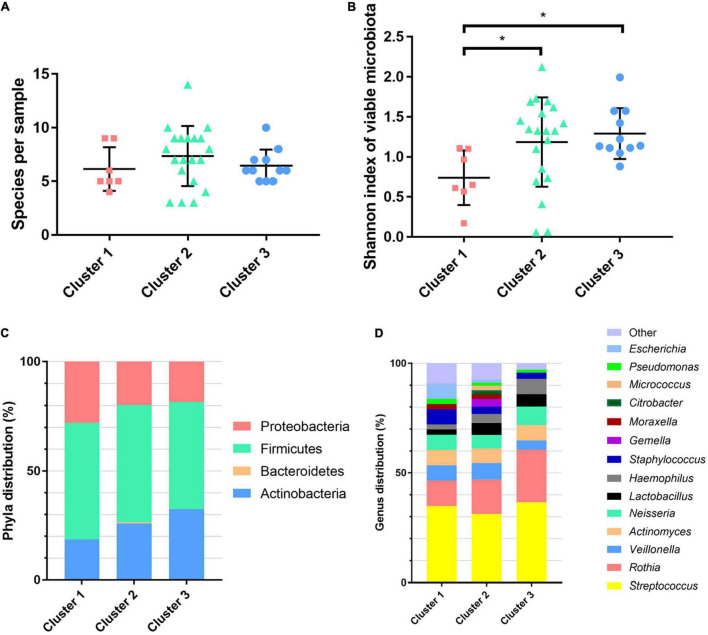
Bacterial diversity and composition of the airway microbiota of the chronic obstructive pulmonary disease patients from the three clusters. **(A)** Number of species per sample. **(B)** Alpha diversity of viable microbiota: Shannon index. **(C)** Phylum distribution and **(D)** genus distribution. *p < 0.05 using Kruskal–Wallis test with Dunn’s multiple-comparison test.

The number of bacteria identified did not differ depending on the clusters [cluster 1: mean of 6.1 species per sample (*n* = 7), cluster 2: mean of 7.4 species per sample (*n* = 20), and cluster 3: mean of 6.5 species per sample (*n* = 11); *p* = 0.34] (Figure 2A and Supplementary Table 2). Cluster 1 was characterized by a lower α-diversity when compared with clusters 2 and 3 (*p* = 0.035 and 0.0379, respectively; Figure 2B). The predominant phyla were similar in the three clusters (Firmicutes, Proteobacteria, and Actinobacteria; Figure 2C), and the different genera had the same repartition, with a predominance of *Streptococcus*, *Rothia*, *Veillonella*, and *Actinomyces*, representing more than half of the bacteria identified (Figure 2D). Anaerobic bacteria were found in 65.8% (25/38) of patients, mostly *Actinomyces* spp. (16/38) and *Veillonella* spp. (17/38) with a similar repartition between clusters (Supplementary Table 2). The proportion of anaerobic bacteria represented 13.83 ± 11.7% of bacteria in samples (mean ± SD).

**FIGURE 3:**
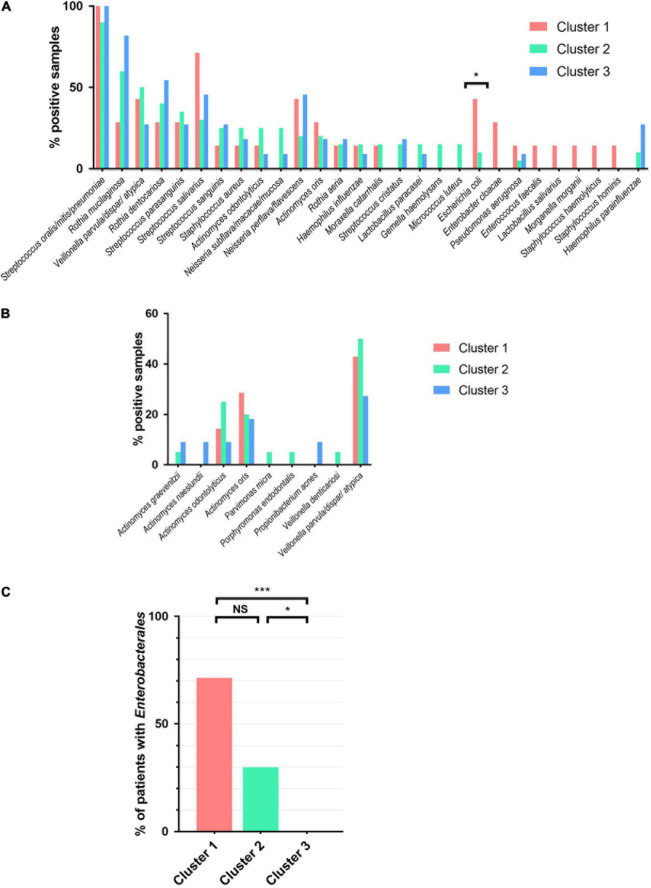
Prevalence of the main bacteria in airway microbiota in the sputa of patients. **(A)** Bacteria prevalence (note: bacteria with less than 10% frequency for the three clusters are not listed). **(B)** Anaerobic bacteria prevalence. **(C)** *Enterobacterales* prevalence (*Citrobacter braakii*, *Citrobacter freundii*, *Citrobacter koseri*, *Enterobacter cloacae*, *Escherichia coli*, *Klebsiella oxytoca*, *Morganella morganii*, *Proteus mirabilis*, and *Raoultella ornithinolytica*). Chi-square test. *p < 0.05, ***p < 0.001. NS, not significant.

We next analyzed the prevalence of the different species in the three clusters (Figures 3A,B for anaerobic bacteria). *Streptococcus oralis/mitis/pneumoniae* was the most common bacteria in all groups, being found in more than 90% of samples. Interestingly, *Escherichia coli* was isolated in sputum of more than 40% patients from cluster 1 and none in cluster 3 (42.9 vs. 0%, *p* = 0.018; Figure 3A). Furthermore, we identified that *Enterobacterales*, to which *E. coli* belongs, was more prevalent in cluster 1 than in cluster 3 (71.4 vs. 0%, *p* = 0.001). Patients belonging to cluster 2 showed an intermediate rate of *Enterobacterales* carriage at 30% (*p* = 0.04 vs. cluster 3 and not significant vs. cluster 1; Figure 3C). The 14 *Enterobacterales* described in 11 patients (28.9%) belonged to the genera *Escherichia*, *Citrobacter*, *Enterobacter*, *Klebsiella*, *Morganella*, *Proteus*, and *Raoultella* (Supplementary Table 2).

In this cohort of COPD patients at stable state, some PPM were detected: *S. aureus* (*n* = 8, 21.1%), *H. influenzae* (*n* = 5, 13.2%), *M. catarrhalis* (*n* = 4, 10.5%) and *P. aeruginosa* (*n* = 3, 7.4%), with the same prevalence in the three clusters (Figure 3A and Supplementary Table 2).

The bacterial quantifications of the microbiota ranged from 10^2^ to 10^9^ CFU/ml, with a median of 10^6^ CFU/ml. The bacterial load was higher in cluster 1 when compared with clusters 2 and 3 (median 1.0 × 10^7^ vs. 7.8 × 10^5^ and 1.0 × 10^5^ CFU/ml, *p* = 0.012 and 0.0007, respectively; Supplementary Table 2). The loads of *Enterobacterales* were mostly low, as 71.4% (10/14) were present at less than 10^7^ CFU/ml (median of 10^6^ CFU/ml).

Therefore, based on airway microbiota analysis, we identified a cluster of stable COPD patients that was enriched in *Enterobacterales* and characterized by decreased α-diversity and higher median bacterial load.

### Relationship Between the Airway Microbiota and Clinical Data

**FIGURE 4:**
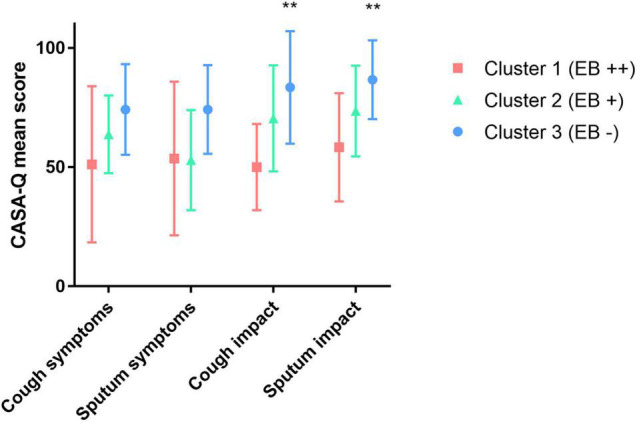
Relationship between airway microbiota and CASA-Q score in chronic obstructive pulmonary disease patients. EB, Enterobacterales.**p < 0.001 (chi-square test between clusters 1 and 3).

To determine if cluster 1 was further characterized by distinct phenotypic features, we next underwent an in-depth analysis of clinical, functional, and morphological characteristics of the patients depending on the clusters (Table 1). Taking into account the *Enterobacterales* distribution within the clusters and the limited number of patients, we compared cluster 1 (71.4% *Enterobacterales*) to cluster 3 (0% *Enterobacterales*). Cluster 2 was an intermediate stage between clusters 1 and 3 with 30% of carriage. Clusters 1 and 3 did not differ in terms of demography, lung function, emphysema score, exacerbations in the last year, or inhaled treatment. When compared with cluster 3, patients in cluster 1 had a more frequent cough (100 vs. 54.5%, *p* = 0.036); this was concordant with the trend observed in the CASA-Q score for cough symptoms (*p* = 0.087). Cluster 2 showed an intermediate rate of cough at 80%. In addition, the impact of both cough and sputum (CASA-Q) was more pronounced in cluster 1 when compared with cluster 3 (*p* < 0.01; Figure 4). Patients in cluster 1 had significantly more pronounced anxiety scores and altered mental health (SF-36) than patients in cluster 3.

## Discussion

Although culture-independent sequencing-based techniques unraveled part of the microbiome not detectable by conventional culturing methods, like not readily cultivable bacteria, they are currently still difficult to implement in clinical routine diagnostics in a cost- and time-efficient way (Han et al., 2012; Ditz et al., 2020). Moreover, the advent of new methods of bacterial identification using mass spectrometry (MALDI-TOF) has revolutionized the microbiological laboratory practices, bringing a simple, very fast, and low-cost way to identify numerous bacteria in patient samples compared to previous phenotypical identifications (Croxatto et al., 2012; Dingle and Butler-Wu, 2013). Then, an extended cultivation approach, a traditional culture-based strategy using additional media than those commonly used for sputum analysis, could be a way to sharpen the airway microbiota for patients in the everyday practice of clinical microbiology laboratories (Sibley et al., 2011). In this study, we described, for the first time to our knowledge, the viable airway microbiota of stable COPD patients using an extended culture-based strategy of sputum.

Based on this airway microbiota analysis, we identified a cluster of stable COPD patients whose samples were enriched in *Enterobacterales*, characterized by loss of microbiota diversity and higher median bacterial load, and who exhibited a distinct clinical phenotype, including predominant cough and respiratory symptom-associated impact on mental health.

Analysis of the airway microbiota by PCoA found three distinct clusters in our cohort of stable COPD patients. They showed no differences in proportion of the phylum, all dominated by Firmicutes, Proteobacteria, and Actinobacteria, as previously described in other reports analyzing the microbiota of stable COPD patients (Garcia-Nuñez et al., 2014; Einarsson et al., 2016; Wang et al., 2018; Tangedal et al., 2019; Yang et al., 2021). Similar to other microbiome studies in stable COPD patients (Wilkinson et al., 2003; Garcia-Nuñez et al., 2014; Aguirre et al., 2015; Einarsson et al., 2016; Wang et al., 2018; Tangedal et al., 2019; Dicker et al., 2021; Yang et al., 2021), the vast majority of bacteria belonged to the genera *Streptococcus*, *Rothia*, *Veillonella*, and *Actinomyces*, in the same proportion as in the three clusters. We also found some PPM, such as *S. aureus*, *H. influenzae*, *M. catarrhalis*, and *P. aeruginosa*, in proportions quite similar to those previously described in stable patients (Marin et al., 2009; Garcia-Nuñez et al., 2014; Aguirre et al., 2015; Einarsson et al., 2016; Leung et al., 2017), and the prevalence was identical in the three clusters. *P. aeruginosa* was scarce in stable COPD patients (only 7.4% in our study), while the literature reports a prevalence of up to 29.4% (Garcia-Nuñez et al., 2014; Aguirre et al., 2015; Einarsson et al., 2016; Leung et al., 2017). As previously described by Murphy et al. (2008) it may indicate the clearance of this pathogen between exacerbations, conversely to CF where *P. aeruginosa* is likely a chronic lung colonizer (O’Sullivan and Freedman, 2009).

There is a growing body of evidence that suggests a potential role of anaerobes in the pathogenesis of CF (Lamoureux et al., 2019; Thornton and Surette, 2021). Since there is a lack of literature for COPD, we decided to translate such an issue to this other chronic respiratory disease. While strict anaerobic bacteria are not cultured in the conventional processing of sputum (Leber, 2019), we attempted to identify anaerobes, with a particular interest in *Porphyromonas* spp. Indeed Diao et al. (2018) who described microbiota in a stable COPD cohort, also reported a correlation between the presence of *P. catoniae* and the weaker severity of the symptoms. Interestingly, similar findings have been recently shown in CF wherein *Porphyromonas catoniae* has been proposed as a potential predictive biomarker of *P. aeruginosa* pulmonary infection (Keravec et al., 2019). Although we were able to isolate anaerobes in 65% of patients, we found only one positive sputum with *Porphyromonas* spp. (*Porphyromonas endodontalis*) from a *P. aeruginosa* non-carrier patient (Supplementary Table 2).

Interestingly, we found a higher prevalence of *Enterobacterales* in cluster 1 compared to cluster 3 (71.4 vs. 0%), including *E. coli* (42.9 vs. 0%), which is the leader of this important bacterial order. Most of *Enterobacterales* (71.4%) were found below 10^7^ CFU/ml, which is the threshold to define a positive culture in sputum (Wilson and Martin, 1972; Kalin et al., 2015). *Enterobacterales* are the main component of the gut facultative aerobic microbiota and are not usually found in airway microbiota (Donaldson et al., 2016; Dekaboruah et al., 2020). These bacteria are scarcely isolated in exacerbated COPD patients (mainly in advanced disease when observed), and their pathogenicity remains undefined (Sethi and Murphy, 2008; Marin et al., 2009; Domenech et al., 2013; Matkovic and Miravitlles, 2013; Martinez et al., 2014). In stable COPD patients, they have also been rarely reported, with the same unclear role (Sethi and Murphy, 2008). It is worth noting that, on the few studies reporting the presence of *Enterobacterales* in stable patients, most of them used a culture-based approach (Marin et al., 2009; Aguirre et al., 2015). Aguirre et al. (2015) isolated *Enterobacterales* in 7/19 of their patients, but surprisingly the sequencing approach used to be compared with the culture approach found only 1/19 patients with *Enterobacterales*. The authors concluded a probable overestimation of these genera by culture, but their lack of detection by sequencing methods seems questionable since molecular detection is known to be more sensitive than culture-based approach (Han et al., 2012; Ditz et al., 2020). As in our study, loads of *Enterobacterales* were mostly under 10^7^ CFU/ml (8/9 strains) (Aguirre et al., 2015). Besides the presence of *Enterobacterales*, cluster 1 was also characterized by a decrease in α-diversity and a greater bacterial load of 10^7^ CFU/ml, which is two more logs than cluster 3. Interestingly, the increase of airway bacterial load and loss of diversity at a stable state have been previously linked to more severe airflow limitation (FEV_1_ decline) and severity of the disease (Wilkinson et al., 2003; Garcha et al., 2012; Galiana et al., 2013; Garcia-Nuñez et al., 2014). In our study, cluster 1 was not characterized by a more severe airflow limitation, but with a clinically relevant phenotype associating cough and respiratory symptom-associated psychological impact on mental health. The impact of PPM colonization on daily respiratory symptoms (breathlessness, cough, and sputum) (Desai et al., 2014) and of chronic cough on depression and anxiety (Deslee et al., 2016) has previously been reported. In addition, chronic cough has been identified as a risk factor for poor outcomes in COPD patients (Miravitlles, 2011), including the onset of exacerbations. Whether the microbiome-associated phenotype that we identified is at a higher risk of exacerbations or accelerated lung function decline will require longitudinal studies. In addition, whether *Enterobacterales* have a direct impact on symptoms and whether its eradication would benefit the symptom-associated mental health of the patient have to be studied.

In the light of such questioning, it may be further taken into account to change the sputum analytical process in our clinical microbiology laboratories to track *Enterobacterales* correctly. As for CF, low bacterial loads could be clinically relevant as highlighted here with the cluster 1 results. Therefore, bacterial culture would be performed with serial dilutions (e.g., up to 1/100,000) of the sputum, allowing it to reach lower thresholds than the currently recommended 10^7^ CFU/ml (Wilson and Martin, 1972; Kalin et al., 2015). Moreover, the use of Gram-negative-bacillus-selective agar (such as MacConkey) and/or Gram-negative-bacillus-chromogenic agar would be helpful to better detect the subdominant *Enterobacterales* species within the non-pathogenic dominant microbiota. Despite other studies focusing on microbiome characterization, which reported the presence of *Enterobacterales* in the airway microbiota of COPD patients, we did not find any studies addressing whether this order could be associated with worse respiratory symptoms. Debates of the CF community about the pathogenicity of *Enterobacterales* could be taken into account to help filling this gap in COPD—for instance, recent studies reported that *Enterobacterales* were evidenced in CF patients with a prevalence of *E. coli* that could reach 25% (Gilligan, 1991; Barillova et al., 2014; Izydorczyk et al., 2020; Vermeulen et al., 2020). Some authors reported the isolation of *Enterobacterales* preceding *P. aeruginosa* and its association with worse lung function (Vermeulen et al., 2020). It is worth noticing that all of them tracked *Enterobacterales* below the 10^7^CFU/ml threshold (Barillova et al., 2014; Izydorczyk et al., 2020; Vermeulen et al., 2020).

Our study has potential limitations, such as the limited number of patients included, the one-point sampling, and the potential lack of technical sensitivity for anaerobes. First, future studies with larger cohorts are needed to confirm the trend observed, with longitudinal follow-up studies, regarding the stability of the *Enterobacterales* carriage, the impact on airway inflammation, and the number of exacerbations. Second, we cannot exclude that the limited detection of slow-growing or anaerobic bacteria may result from technical limitations inherent to the conventional culture-based approach. We did not want to change frameworks for sampling by the physicians, so we did not use any collecting device that could ensure the preservation of an anaerobic atmosphere for the sputa until processing in the laboratory (Lamoureux et al., 2021). Moreover, we did not compare our results obtained by culture methods with sequencing-based data, as we only focused on culture methods applicable in most clinical microbiology laboratories. Eventually, MALDI-TOF identification method is unsuitable to accurately detect *Streptococcus pneumoniae* (Farfour et al., 2020), which precluded studying this species specifically in this work.

In conclusion, we analyzed the viable airway microbiota of stable COPD patients by a culture-based approach. We described a phenotype of patients associated with *Enterobacterales* and higher bacterial load, characterized by predominant cough and respiratory symptom-associated impact on mental health. This impact needs to be determined in future studies in order to clarify whether it could improve the clinical management of COPD patients. If confirmed, it will encourage to modify the processing of COPD sputum in the everyday practice of clinical microbiology laboratories.

## Data Availability Statement

The original contributions presented in the study are included in the article/Supplementary Material, further inquiries can be directed to the corresponding author/s.

## Ethics Statement

The studies involving human participants were reviewed and approved by the Regional Ethics Committee (Comité de Protection des Personnes—Dijon EST I, N°2016-A00242-49) and referenced as NCT02924818 in clinical trials. The patients/participants provided their written informed consent to participate in this study.

## Author Contributions

TG, AM, J-MP, and GD conducted the study design and revised the manuscript. J-MP, SD, CL, JA, PM, and GD collected samples and gathered clinical information from the subjects. AM, CC, and TG conducted the microbiological analysis. AB performed the PCoA analysis. All authors contributed toward data analysis, drafting, and revising the manuscript and declared their contributions to this manuscript.

## Conflict of Interest

The authors declare that the research was conducted in the absence of any commercial or financial relationships that could be construed as a potential conflict of interest.

## Publisher’s Note

All claims expressed in this article are solely those of the authors and do not necessarily represent those of their affiliated organizations, or those of the publisher, the editors and the reviewers. Any product that may be evaluated in this article, or claim that may be made by its manufacturer, is not guaranteed or endorsed by the publisher.
