# Supplementary material for: Extended Bacteria Culture-Based Clustering Identifies a Phenotype Associating Increased Cough and Enterobacterales in Stable Chronic Obstructive Pulmonary Disease
Source: Front Microbiol. 2021 Dec 14;12:781797. doi: 10.3389/fmicb.2021.781797 (PMC8712763; doi:10.3389/fmicb.2021.781797)
Supplement: Supplementary file 1 [file Data_Sheet_1.docx]

Supplementary Material

# Supplementary Tables

**Table S1 : Extended culture protocole**

| **Media** | **No dilution** | **Dilution 1/1000** | **Dilution 1/10000** | **Dilution 1/100000** | **Atmosphere** |
| --- | --- | --- | --- | --- | --- |
| Columbia blood agar | x | x | x | x | Aerobic 48h |
| Chocolate agar | x | x | x | x | 5% CO_2_ 48h |
| Schaedler agar | x |  | x |  | Anaerobic 5 days |
| Cetrimide agar | x |  |  |  | Aerobic 48h |

**Table S2: Prevalence and quantification of the bacteria in airway microbiota of COPD patients**

| **Cluster 1** | **n=7** | **%** | **Quanti-fication (median)** | **Cluster 2** | **n=20** | **%** | **Quanti-fication (median)** | **Cluster 3** | **n=11** | **%** | **Quanti-fication (median)** |
| --- | --- | --- | --- | --- | --- | --- | --- | --- | --- | --- | --- |
| *Streptococcus oralis/mitis/pneumoniae* | 7 | 100,0 | 1,00E+08 | *Streptococcus oralis/mitis/pneumoniae* | 18 | 90,0 | 1,00E+06 | *Streptococcus oralis/mitis/pneumoniae* | 11 | 100,0 | 1,00E+06 |
| *Streptococcus salivarius* | 5 | 71,4 | 1,00E+07 | *Rothia mucilaginosa* | 12 | 60,0 | 1,00E+06 | *Rothia mucilaginosa* | 9 | 81,8 | 1,00E+05 |
| *Veillonella parvula/dispar/ atypica* | 3 | 42,9 | 1,00E+05 | *Veillonella parvula/dispar/ atypica* | 10 | 50,0 | 1,00E+05 | *Rothia dentocariosa* | 6 | 54,5 | 1,00E+05 |
| *Escherichia coli* | 3 | 42,9 | 1,00E+05 | *Rothia dentocariosa* | 8 | 40,0 | 5,50E+05 | *Neisseria perflava/flavescens* | 5 | 45,5 | 1,00E+05 |
| *Neisseria perflava/flavescens* | 3 | 42,9 | 1,00E+08 | *Streptococcus parasanguinis* | 7 | 35,0 | 1,00E+06 | *Streptococcus salivarius* | 5 | 45,5 | 5,00E+05 |
| *Enterobacter cloacae* | 2 | 28,6 | 5,50E+05 | *Streptococcus salivarius* | 6 | 30,0 | 1,00E+06 | *Haemophilus parainfluenzae* | 3 | 27,3 | 1,00E+05 |
| *Rothia mucilaginosa* | 2 | 28,6 | 1,00E+06 | *Actinomyces odontolyticus* | 5 | 25,0 | 1,00E+05 | *Veillonella parvula/dispar/ atypica* | 3 | 27,3 | 1,00E+05 |
| *Streptococcus parasanguinis* | 2 | 28,6 | 5,05E+07 | *Streptococcus sanguinis* | 5 | 25,0 | 1,00E+07 | *Streptococcus parasanguinis* | 3 | 27,3 | 1,00E+06 |
| *Actinomyces oris* | 2 | 28,6 | 7,50E+07 | *Staphylococcus aureus* | 5 | 25,0 | 1,00E+04 | *Streptococcus sanguinis* | 3 | 27,3 | 1,00E+05 |
| *Rothia dentocariosa* | 2 | 28,6 | 1,00E+07 | *Neisseria subflava/macacae/mucosa* | 5 | 25,0 | 1,00E+06 | *Streptococcus cristatus* | 2 | 18,2 | 6,50E+05 |
| *Morganella morganii* | 1 | 14,3 | 5,00E+06 | *Actinomyces oris* | 4 | 20,0 | 1,00E+05 | *Staphylococcus aureus* | 2 | 18,2 | 5,50E+04 |
| *Staphylococcus haemolyticus* | 1 | 14,3 | 1,00E+07 | *Neisseria perflava/flavescens* | 4 | 20,0 | 5,05E+05 | *Actinomyces oris* | 2 | 18,2 | 1,00E+05 |
| *Enteroccocus faecalis* | 1 | 14,3 | 1,00E+04 | *Haemophilus influenzae* | 3 | 15,0 | 1,00E+07 | *Rothia aeria* | 2 | 18,2 | 5,01E+05 |
| *Rothia aeria* | 1 | 14,3 | 1,00E+07 | *Lactobacillus paracasei* | 3 | 15,0 | 1,00E+04 | *Lactobacillus rhamnosus* | 1 | 9,1 | 5,00E+04 |
| *Staphylococcus hominis* | 1 | 14,3 | 1,00E+07 | *Rothia aeria* | 3 | 15,0 | 1,00E+06 | *Actinomyces odontolyticus* | 1 | 9,1 | 1,00E+05 |
| *Haemophilus influenzae* | 1 | 14,3 | 5,00E+07 | *Gemella haemolysans* | 3 | 15,0 | 1,00E+07 | *Streptococcus mutans* | 1 | 9,1 | 1,00E+05 |
| *Streptococcus sanguinis* | 1 | 14,3 | 1,00E+09 | *Moraxella catarrhalis* | 3 | 15,0 | 1,00E+06 | *Pseudomonas aeruginosa* | 1 | 9,1 | 1,00E+02 |
| *Lactobacillus salivarius* | 1 | 14,3 | 1,00E+07 | *Streptococcus cristatus* | 3 | 15,0 | 1,00E+07 | *Stenotrophomonas maltophilia* | 1 | 9,1 | 1,00E+06 |
| *Moraxellacatarrhalis* | 1 | 14,3 | 5,00E+07 | *Micrococcus luteus* | 3 | 15,0 | 1,00E+04 | *Lactobacillus fermentum* | 1 | 9,1 | 5,00E+04 |
| *Staphylococcus aureus* | 1 | 14,3 | 1,00E+04 | *Haemophilus parainfluenzae* | 2 | 10,0 | 1,00E+06 | *Streptococcus gordonii* | 1 | 9,1 | 1,00E+06 |
| *Actinomyces odontolyticus* | 1 | 14,3 | 1,00E+05 | *Gemella sanguinis* | 2 | 10,0 | 5,50E+04 | *Lactobacillus paracasei* | 1 | 9,1 | 1,00E+04 |
| *Pseudomonas aeruginosa* | 1 | 14,3 | 1,00E+03 | *Lactobacillus fermentum* | 2 | 10,0 | 5,50E+03 | *Haemophilus influenzae* | 1 | 9,1 | 1,00E+05 |
|  | Median | | 1,00E+07 | *Granulicatella adiacens* | 2 | 10,0 | 5,01E+06 | *Actinomyces naeslundii* | 1 | 9,1 | 1,00E+06 |
|  |  |  |  | *Streptococcus gordonii* | 2 | 10,0 | 5,05E+06 | *Lactobacillus plantarum* | 1 | 9,1 | 1,00E+05 |
|  |  |  |  | *Escherichia coli* | 2 | 10,0 | 1,00E+03 | *Haemophilus parahaemolyticus* | 1 | 9,1 | 1,00E+05 |
|  |  |  |  | *Veillonella denticariosi* | 1 | 5,0 | 1,00E+04 | *Neisseria subflava/macacae/mucosa* | 1 | 9,1 | 1,00E+04 |
|  |  |  |  | *Lactococcus lactis* | 1 | 5,0 | 1,00E+05 | *Actinomyces graevenitzii* | 1 | 9,1 | 1,00E+06 |
|  |  |  |  | *Citrobacter koseri* | 1 | 5,0 | 1,00E+06 | *Propionibacterium acnes* | 1 | 9,1 | 1,00E+05 |
|  |  |  |  | *Citrobacter freundii* | 1 | 5,0 | 1,00E+06 |  | Median | | 1,00E+05 |
|  |  |  |  | *Lactobacillus delbrueckii* | 1 | 5,0 | 1,00E+05 |  |  |  |  |
|  |  |  |  | *Citrobacter braakii* | 1 | 5,0 | 1,00E+07 |  |  |  |  |
|  |  |  |  | *Lactobacillus gasseri* | 1 | 5,0 | 1,00E+05 |  |  |  |  |
|  |  |  |  | *Streptococcus constellatus* | 1 | 5,0 | 1,00E+07 |  |  |  |  |
|  |  |  |  | *Corynebacterium propinquum* | 1 | 5,0 | 1,00E+06 |  |  |  |  |
|  |  |  |  | *Lactobacillus salivarius* | 1 | 5,0 | 1,00E+05 |  |  |  |  |
|  |  |  |  | *Streptococcus agalactiae* | 1 | 5,0 | 1,00E+04 |  |  |  |  |
|  |  |  |  | *Parvimonas micra* | 1 | 5,0 | 1,00E+04 |  |  |  |  |
|  |  |  |  | *Haemophilus parahaemolyticus* | 1 | 5,0 | 1,00E+05 |  |  |  |  |
|  |  |  |  | *Porphyromonas endodontalis* | 1 | 5,0 | 5,00E+04 |  |  |  |  |
|  |  |  |  | *Streptococcus mutans* | 1 | 5,0 | 1,00E+06 |  |  |  |  |
|  |  |  |  | *Proteus mirabilis* | 1 | 5,0 | 1,00E+03 |  |  |  |  |
|  |  |  |  | *Klebsiella oxytoca* | 1 | 5,0 | 1,00E+07 |  |  |  |  |
|  |  |  |  | *Pseudomonas aeruginosa* | 1 | 5,0 | 1,00E+07 |  |  |  |  |
|  |  |  |  | *Streptococcus salivarius/vestibularis* | 1 | 5,0 | 1,00E+05 |  |  |  |  |
|  |  |  |  | *Pseudomonas koreensis* | 1 | 5,0 | 1,00E+03 |  |  |  |  |
|  |  |  |  | *Streptococcus vestibularis* | 1 | 5,0 | 1,00E+06 |  |  |  |  |
|  |  |  |  | *Raoultella ornithinolytica* | 1 | 5,0 | 1,00E+07 |  |  |  |  |
|  |  |  |  | *Bifidobacterium dentium* | 1 | 5,0 | 1,00E+03 |  |  |  |  |
|  |  |  |  | *Actinomyces graevenitzii* | 1 | 5,0 | 1,00E+06 |  |  |  |  |
|  |  |  |  | *Moraxella osloensis* | 1 | 5,0 | 1,00E+03 |  |  |  |  |
|  |  |  |  |  | Median | | 7,75E+05 |  |  |  |  |
